# Eye movements, attention, and expert knowledge in the observation of Bharatanatyam dance

**DOI:** 10.16910/jemr.11.2.11

**Published:** 2018-12-28

**Authors:** Raganya Ponmanadiyil, Matthew H. Woolhouse

**Affiliations:** McMaster University, Canada

**Keywords:** Bharatanatyam, dance, music, eye tracking, fixations, attention, expertise

## Abstract

Previous research indicates that dance expertise affects eye-movement behaviour—dance experts tend to have faster saccades and more tightly clustered fixations than novices when observing dance, suggesting that experts are able to predict movements and process choreographic information more quickly. Relating to this, the present study aimed to explore (1) the effects of expertise on eye movements (as a proxy for attentional focus and the existence of movement-dance schemas) in Indian Bharatanatyam dance, and (2) narrative dance, which is an important component of Bharatanatyam. Fixation durations, dwell times, and fixation-position dispersions were recorded for novices and experts in Bharatanatyam (N = 28) while they observed videos of narrative and non-narrative Bharatanatyam dance. Consistent with previous research, experts had shorter fixation durations and more tightly clustered fixations than novices. Tighter clustering of fixations was also found for narrative dance versus non-narrative. Our results are discussed in relation to previous dance and eye-tracking research.

## Introduction

Dance has long been recognized as a universal, non-verbal communicative medium through which emotional states, social narratives, and myths may be conveyed(1). Evidence from dance and emotion research suggests that humans are highly attuned to dancers’ affective states, communicated through body and limb movements; for overview(2). For many world cultures, music and dance are not separable cultural categories; for example, Merriam 1964 quotes Gbeho as stating that in the indigenous music of the Gold Coast “If we speak of a man being musical we mean that he understands all the dances, the drums and the songs”(3). From a global, cultural perspective, dance is frequently integrated into ritualistic and religious ceremonies, both in contemporary societies(4) and historically(5). Moreover, dance is considered an essential ingredient in the formation and maintenance of group identities(6, 7, 8). For example, researchers investigated the effects of an instructional program in creative dance on the social development of preschool children(9). Teachers and parents’ ratings of the children's social skills, both before and after the program, revealed significant gains in social competence, suggesting that creative dance instruction for (at-risk) preschoolers improves social interactions and behaviour(9). Therefore, dance might exist today due to an evolutionary past in which it promoted prosocial behaviour, increasing one’s ability to survive within ‘primitive’ communities(10, 11, 12, 13, 14).

Key to dance’s ability to engender social affiliation is, arguably, its capacity to direct the attentional foci and cognitive resources of individuals. Evidence for this, and its effect upon interpersonal memory, was investigated(15). In brief, the study required untrained dancers to recall various attributes of one another after having danced in groups, some synchronously, others asynchronously(15). Results showed that those who danced together in time were more likely to remember each other. It was hypothesized that this may facilitate social bonding, which would presumably be difficult to achieve in situations where interpersonal memory was absent(15). For recent related research regarding dance and social bonding, see(16).

In a related study, eye movements of participants observing pairs of dancers, one of whom danced in synchrony with a musical track, while the other danced asynchronously was investigated(17). Gaze dwell-times amongst participants were significantly greater for the music-synchronous dancer, indicating a possible mechanism through which attention may have been directed towards the in-tempo dancers in the group study(15). Moreover, they investigated fixations across different body regions, including head, torso, legs and feet(17). Perhaps, paradoxically, given the importance of legs and feet in most dancing, feet attracted significantly less dwell time than any other body region. In sum, dance, in combination with music, appears to have the ability to direct attention as detected in the eye movements of observers. This is on par with everyday motion, which also produces gaze toward the head(18).

Two types of eye movements are usually studied in scene-perception research: fixations, during which the eyes remain still and new information is acquired from the visual field, and saccades, movements between fixations during which vision is suppressed and no new information is gained(19). In reading research, regressions—reverse saccades in which the eyes backtrack to the previous fixation point—are frequently examined in relation to syntactic comprehension(20, 21). In scene perception, fixations are usually between 260–330 ms, interspersed with saccades lasting about 50 ms(19). Saccade lengths can differ significantly depending on the type of image being viewed, and are about 40% longer for complex natural scenes than abstract patterns(22).

While several individuals may observe the same dance, various studies indicate that *how* each individual completes this potentially cognitively demanding task depends upon context and experience. For instance, researchers found that emotional arousal increases upon repeated exposure to a musical piece, suggesting that, in general, familiarity influences cognition, possibly by allowing individuals to develop schematic expectations(23); see also(24). And while, since the early 1970’s, it has been known that expertise and familiarity of the static visual stimuli significantly influences eye movements(25), it was only in the 1990’s that researchers thoroughly investigated how expectations influence human motion perception. For example, individuals’ schema of biological motion guides them to fixate on the ends of limbs to track the movement of human extremities(18).

With respect to dance, in a study examining the influence of expertise on the observation of dance, it was found that people with advanced dance training had shorter fixation durations and faster saccades than novices(26). In their analysis of body-directed fixations, the experienced choreographer in the study attended mostly to the head of the dancers, while novices attended equally to the head, neck, torso and arms(26). It was suggested that the eye movements of the expert in their study was “likely guided by the expectancies and schemata in long-term memory”, and that this was due to them being “adept at abstracting and extracting key information from complex movement material” (p. 23)(26). Which is to say, fixations and saccades are influenced by the type of image being viewed and expertise that, in turn, are suggestive of viewers’ underlying cognitive processes, attentional foci, and schematic knowledge. The differences between experts and novices that were found(26) are consistent with earlier findings that found the performance of expert dancers on a dance sequence recall task depended in part on the amount of structure in the material, which implied that subject’s knowledge base impacted memorization(27). For related neurological research see(28).

Common to the work of (17, 26) and others(29) is the treatment of dance as a relatively abstract form of human movement, seemingly divorced from its richer cultural setting in which, for example, narrative meaning may be conveyed; although, see(30) for the effect of culture on eye movements during scene perception. The influence that narrative contexts can have on perception, and, in particular, eye movements, has recently received increased attention. Researchers studied the relationship between film viewers’ eye movements and their comprehension of film narrative by investigating whether eye movements differed based on understanding(31). Referred to as the *mental model* hypothesis, this notion is distinct from the alternative *tyranny of film* hypothesis, which stipulates that differences due to understanding are overwhelmed by viewers’ attentional synchrony(31).

In brief, two groups were presented with a short clip from a James Bond movie in which a villain (“Jaws”) was about to fall from the sky onto a circus tent(31). Critically, one group saw only the clip while the other saw the preceding two-and-a-half minutes of the movie(31). The researchers hypothesized that the second group, who viewed the clip with its narrative context, would be better able to draw critical inferences and have more coherent perceptions than the group who viewed only the short clip(31). However, despite the difference in the stimuli, both groups showed strong attentional synchrony, and only small between-group variance(31). Overall, then, the results were more consistent with the *tyranny of film* hypothesis than the *mental model* hypothesis, suggesting that narrative context may contribute less to eye movements than visual features such as flicker and motion (i.e. temporal contrast) during free-viewing of videos(31); see also(32, 33).

Our intention in this eye-tracking study was to develop some of the research discussed above in an experiment that examined the effects of expertise and dance narrative on eye movements—that is, dances which, through various gesture sequences, attempt to convey specific, real-world and/or religious meanings. To preempt our hypotheses somewhat, we envisaged that the difference between experts and novices would lead to differences in eye movements. One such dance that lends itself particularly well to this is Indian Bharatanatyam dance. As discussed above, while prior eye-tracking studies have identified several factors that influence the processing of dance—such as expertise—these factors have yet to be explored within broader cultural contexts. Our study sought to provide an expanded cultural understanding of the effects of expertise on observing dance using videos of Bharatanatyam.

### Bharatanatyam

Originating in the southern states of India, Bharatanatyam is an ancient form of female classical dance that involves extensive formal training, passed from teacher to student through years of mentorship, dedication, and practice. The *Natyasastra* scriptures explain Bharatanatyam with reference to a taxonomy of body movements: *nritta* (abstract, ‘pure’ dance, performed without expressing a particular theme or emotion), and *nritya* (representational, interpretive dance, performed to convey emotions and narrative themes); for a detailed explanation see(34). Both *nritta* and *nritya* are produced by a combination of movements and positions involving the feet, limbs, and body, along with hand gestures and facial expressions. These elements constitute the ‘lexicon’ of Bharatanatyam, are highly codified, and are responsible for its distinctive look (along with its brightly coloured, traditional costumes).

One way in which *nritta* and *nritya* can be distinguished is through the facial expressions of the dancers. *Nritta* is predominantly performed with a smile, and, despite eye movements, the face has a fix, somewhat mask-like quality. In *nritya*, multiple dynamic facial expressions can be enacted by the dancer as they portray contrasting emotions, characters and themes. A further distinction is the use of particular hand gestures and shapes, referred to as *hastas* (or sometimes *mudras*). During *nritta*, *hastas* convey no meaning and are entirely decorative. In *nritya*, *hastas* in combination with eye movements and facial expressions can be used to describe objects, communicate concepts (e.g. truth and beauty), and illustrate thoughts, actions, and emotions. In short, within Bharatanatyam there are passages that are comprised entirely of abstract, ‘pure dance’ gestures (*nritta*), whilst others are wholly interpretive and/or representational (*nritya*).

Lastly, although there are different styles of Bharatanatyam, being taught in varying schools, the differences in style lead to only slight variations in rules, forms, and steps. Which is to say, Bharatanatyam conforms to a general set of choreographic rules that span the art form. For example, such requirements include that, in general, a dance step be completed three times, and that movements are executed on the right side of the body before being duplicated on the left. As these rules are common across Bharatanatyam, it can be assumed that dancers with at least five years of training will have an adequate understanding of all the basics movements of Bharatanatyam; however, more experience (e.g. a minimum of eight years) is usually required before an individual is considered to be an expert within the discipline.

### Hypotheses

The present study builds in part upon previous works(17, 26) by using eye-tracking to investigate the following four hypotheses: (1) that experts (of Bharatanatyam) will have shorter fixations than novices, which, if true, would be consistent with the notion that experienced viewers observe dance more efficiently(26); (2) that there will be differences in eye movements while observing narrative dance versus non-narrative dance, and possibly an interaction between the type of dance (narrative versus non-narrative) and expertise, reflecting differences in veridical knowledge; (3) that more fixations (and greater gaze dwell times) will occur in relation to the upper body than lower(17); and (4) that there will be greater attentional similarity between experts than novices due to the influence of shared schematic knowledge concerning Bharatanatyam.

A description of the study’s methods (including participants, stimuli, apparatus, procedure, and analysis), and results now follows.

## Methods

### Participants

28 female undergraduate psychology students and volunteers participated in the study. Participants were categorized into Bharatanatyam experts—individuals possessing at least eight years of formal training—and novices—individuals possessing no training or knowledge of Bharatanatyam. The decision to include only female participants was taken due to a preponderance of females amongst the expert cohort; in order to maintain balance, female participants were therefore also used within the novice cohort. There were 14 experts (mean age = 18.81 years; SD = 1.03) and 14 novices (mean age = 18.92 years; SD = .53). Experts possessed a mean of 9.55 years of Bharatanatyam training (SD = 1.03), and commenced training at about 5 years of age. Novices had no formal training in any dance form, and reported that they had no knowledge of Bharatanatyam, nor had they previously seen it performed. All participants had normal or corrected-to-normal eyesight. Each participant provided informed consent prior to the experiment; student participants were compensated with a single course credit. All procedures involving the participants were consistent with Canadian Tri-Council Policy; the study had ethics clearance from the Research Ethics Board of the host institution.

### Materials

**Figure 1.**
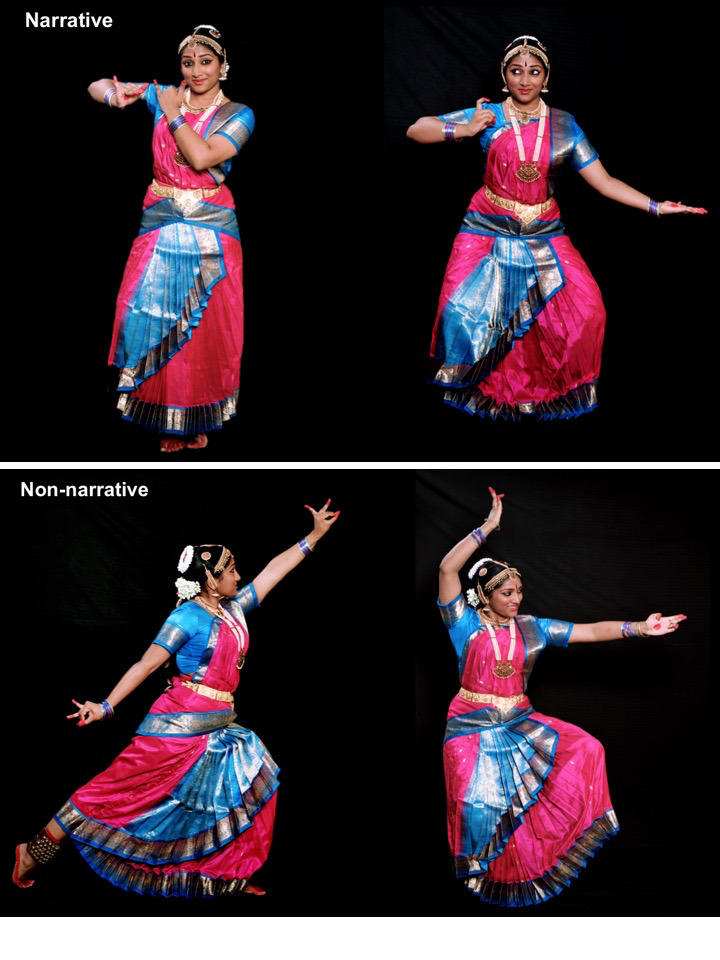
Stills from narrative (*nritya*; top) and non-narrative (*nritta*; bottom) video stimuli.

The primary stimuli for this eye-tracking experiment were taken from a solo Bharatanatyam dance performance (*Arangetram*), presented in front of a live audience by the first author in November 2011. A collection of dance pieces, each ranging in length from 10 to 20 minutes, were selected and trimmed into sixteen video clips, each approximately 30 seconds in duration. Eight of the videos presented Bharatanatyam dance that was narrative in nature (*nritya*), while the remaining eight videos were non-narrative (*nritta*). For the narrative videos, selections were made such that each video portrayed a storyline or specific character. The stage, stage lighting, camera angle, and dancer’s costume were consistent across the video clips; see Figure 1.

Carnatic music, an Indian classical music genre, accompanied the videos and was performed by a small ensemble of musicians to the right of the dancer on stage; however, the ensemble was not visible in the videos. Carnatic music consists of two main elements: *rāga*, the melodic-scalic component of the music, and *tāḷa*, the rhythmic cycles(35). The music used for the narrative and non-narrative had a neutral mood, and did not differ with respect to valence (i.e., was neither overtly positive nor negative in affect). The specific *rāgas*, *tāḷas*, and whether the video was narrative or non-narrative are shown in Table 1.

**Table 1.** Dance title, rāga, and dance type associated with each video (#). The tāḷa for all videos was Adi. The music was composed by Alakananda Nath.

**#**	**Dance title**	**Rāga**	**Dance type**
1	Keerthanam (Hanuman)	Poorvikalyani	Narrative
2	Keerthanam (Hanuman)	Poorvikalyani	Narrative
3	Keerthanam (Hanuman)	Poorvikalyani	Narrative
4	Keerthanam (Hanuman)	Poorvikalyani	Narrative
5	Keerthanam (Hanuman)	Poorvikalyani	Narrative
6	Keerthanam (Hanuman)	Poorvikalyani	Narrative
7	Keerthanam (Hanuman)	Poorvikalyani	Narrative
8	Keerthanam (Hanuman)	Poorvikalyani	Narrative
9	Keerthanam (Kimartham)	Ragamalika	Non-narrative
10	Keerthanam (Kimartham)	Ragamalika	Non-narrative
11	Mangalam	Suruti	Non-narrative
12	Thilana	Hamirkalyani	Non-narrative
13	Thilana	Hamirkalyani	Non-narrative
14	Thilana	Hamirkalyani	Non-narrative
15	Thilana	Hamirkalyani	Non-narrative
16	Thilana	Hamirkalyani	Non-narrative

### Apparatus

Eye movements were recorded using a Mirametrix S2 Eye Tracker at a sampling rate of 60 Hz for each eye; only data recorded from the right eye were used in the subsequent analyses. Blinks were linearly interpolated using the system’s eye-tracking software. The bright-pupil tracking system (sometimes referred to as “red eye effect”, caused by on-camera-axis illumination; see(36), for detailed summary) had a 0.5-degree accuracy range, drift rating of <0.3 degrees, and allowed users to move their heads within the width-height-depth range of 25 × 11 × 30 cm. Video stimuli were presented to participants on a 27” monitor with a resolution of 1920 x 1080. The eye-tracker equipment sat unobtrusively below the monitor, facing the user. An artificially lit booth surrounded the monitor and participant to minimize glare and distraction. Music was presented through AKG K 172 HD headphones, and set to a comfortable level by each participant prior to calibrating the eye-tracker. All participants’ data was exported with the system’s EyeMetrix Software (Mirametrix Inc.).

### Procedure

Participants completed a questionnaire regarding their formal dance and music-training experiences. Following a 9-point eye-tracking calibration process, the 16 video stimuli were presented in a randomized order unique to each participant. A black screen appeared for three seconds between each video. Participants were instructed to observe the dances in no specific manner, but simply to relax and watch the videos as if viewing under normal conditions. Participants were also instructed to tap along to the underlying beat of the music using the computer mouse. This relatively undemanding task ensured that participants attended to both the visual and acoustic/musical elements of the stimuli; in most cases, individuals tend to seek out and move synchronously (and sometimes spontaneously) to an observed beat(37, 38). The video-watching portion of the experiment lasted approximately 12 minutes.

### Analysis

In order to reduce “jitter” and “flicker” effects of the eye-tracking system, and possible artifacts of its data-parsing algorithm, fixations below 100 ms were omitted from the analysis; this resulted in approximately 10% of the data being lost. For discussion on the relative merits of omitting fixation durations below a certain threshold and data-processing algorithms, see(39). In terms of raw data, each participant produced a single data file which contained all their fixation information for all videos. These data files contained the following columns: *Observation number, Frame number, Time stamp, X and Y positions for both Left and Right eyes, and Pupil diametre information.* The frame number and time stamp column were linked such that each frame equated to 16.66 milliseconds (i.e. 60 Hz). The total number of observations (i.e. rows) per participant data file was in the region of 35,000. We requested, from the eye-tracking analytical software, data frames which consisted only of fixations greater than 100 ms (as mentioned above), the x- and y- coordinates, and time stamp information.

Repeated-measure three-way mixed analyses of variance (ANOVA), with Dance-type and Region of Interest (ROI) as within-subject factors, and Expertise as between-subject factor, were run separately on two dependent variables: fixation duration and dwell time. The data frame for these analyses consisted of 112 rows and 7 columns with the following headings: Subject ID; Expertise; Dance-type; ROI; Mean fixation percentage; Fixation duration SD; Dwell time percentage. Within Expertise there were two levels, expert and novice. There were also two levels within Dance-type: narrative and non-narrative. ROI consisted of the screen horizontally divided into two fixed, equally sized regions: top, which covered the dancer’s upper body, and bottom, covering the dancer’s lower body. It should be noted that this split did not absolutely, nor consistently, divide the dancer’s body into two equal parts (i.e. head/torso/arms and hips/legs/feet) due to the movement of the dancer. Expertise and Dance-type with respect to average fixation duration per participant for each factor combination were used to investigate Hypotheses (1) and (2); ROI in relation to percentage dwell time was used to investigate Hypothesis (3).

In order to investigate Hypothesis (4)—that experts will have greater attentional synchrony due to the influence of shared schematic knowledge—each video was divided into overlapping time windows of 1,000 ms, succeeding by 500 ms, producing a total of 60 time windows per video. Fixations were only included in the time windows in which they began, not in subsequent time windows. Thus, if a fixation began in Window 1 and ended in Window 2, its position data was only included in Window 1, not 2. Each fixation was associated with positional coordinates (x, y) with which average SDs for x and y fixation positions were calculated, and then used to calculate average fixation position SDs. The fixation position SDs of each 1,000 ms time window, corresponding to each video, were analyzed using a repeat-measure two-way ANOVA, with *Expertise* as a between-subject factor and *Dance-type* as within-subject factors. Outliers, i.e. a data point outside 1.5 times the interquartile range above the upper quartile and below the lower quartile, were removed from this analysis*.* This resulted in a core data set consisting of 1898 rows and 8 columns with the following headings: *Expertise; Semantics; Video ID; Window ID; Window start time; Average SD, x-axis; Average SD, y-axis; Average SD, x- and y-axes.* This analysis also enabled us to further test Hypothesis (2)—that there will be differences in eye movements while observing narrative versus non-narrative dance.

All data were analyzed using the open-source statistical package R (2.15.0, GUI 1.51). MATLAB (R2014) was used to calculate the fixation SDs per time window per video. Effect sizes are reported with partial eta-squared values.

## Results

### Fixation duration

**Figure 2.**
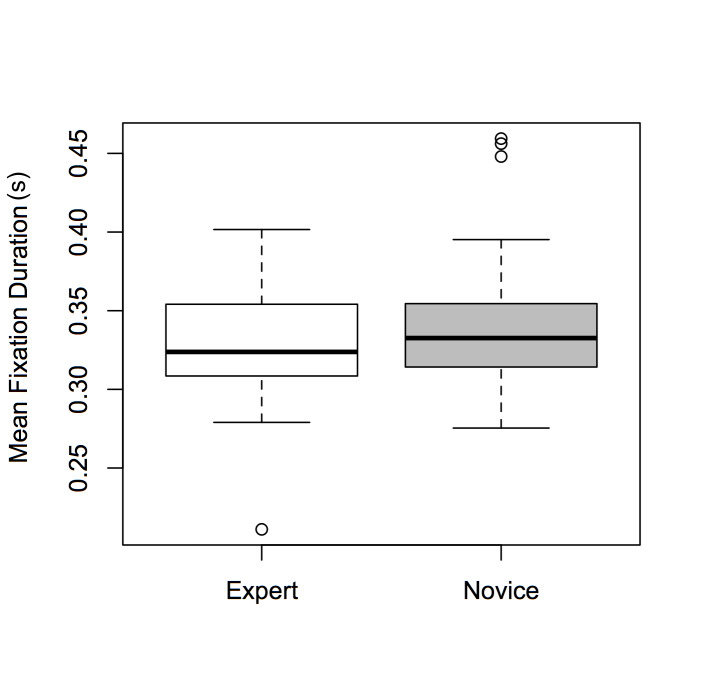
Boxplots of mean fixation durations for *Expertise* (experts and novices).

**Figure 3.**
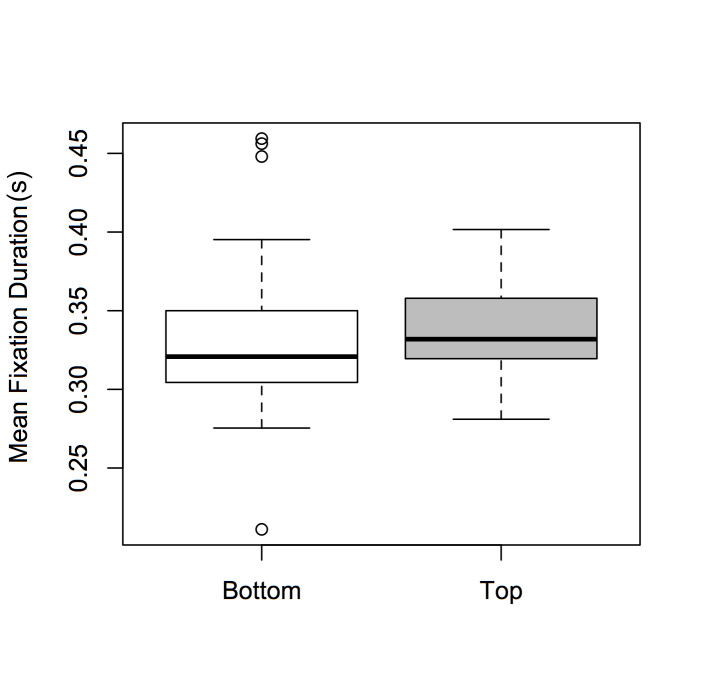
Boxplots of mean fixation durations for *ROI* (bottom and top).

There was a significant main effect of Expertise [F(1,72) = 6.478, p < 0.05, η² = 0.009], and of ROI [F(1,72) = 4.315, p < 0.05, η² = 0.008], but not of Dance-type (F < 1). Expert participants had significantly shorter fixation durations than novices (see Figure 2); participants’ fixation durations were significantly greater when observing the top of the screen versus the bottom (see Figure 3). Whether the dance was narrative or non-narrative had no effect on fixation duration. No significant interactions were found between the factors (F < 1), and excluding outliers did not affect the significance of the results.

### Dwell time

**Figure 4.**
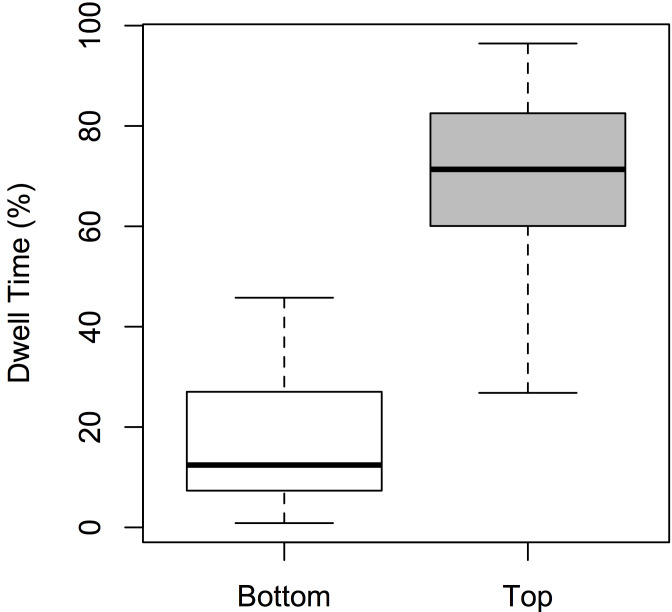
Boxplots of dwell time for *ROI* (bottom and top). Dwell time is expressed as a percentage per participant.

There was a significant main effect of ROI [F(1,72) = 51.424, p < 0.001; η² = 0.054], but not of Expertise or Dance-type (F < 1). Participants spent significantly more time observing the top part of the screen (see Figure 4), irrespective of whether they were experts or novices, or whether the dance was narrative or non-narrative in nature. The non-significant result for Expertise and Dance-type was not surprising given that experts and novices viewed all narrative and non-narrative videos for an equal length of time. No significant interactions were found between the factors (F < 1), and excluding outliers did not affect the significance of the results.

### Fixation position SD

**Figure 5.**
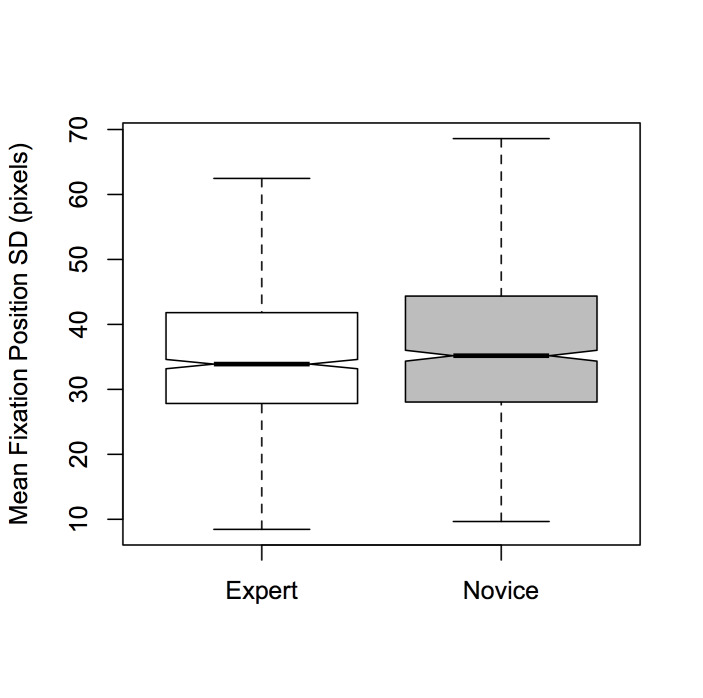
Notched boxplots of mean fixation position SD per time window for *Expertise* (experts and novices).

**Figure 6.**
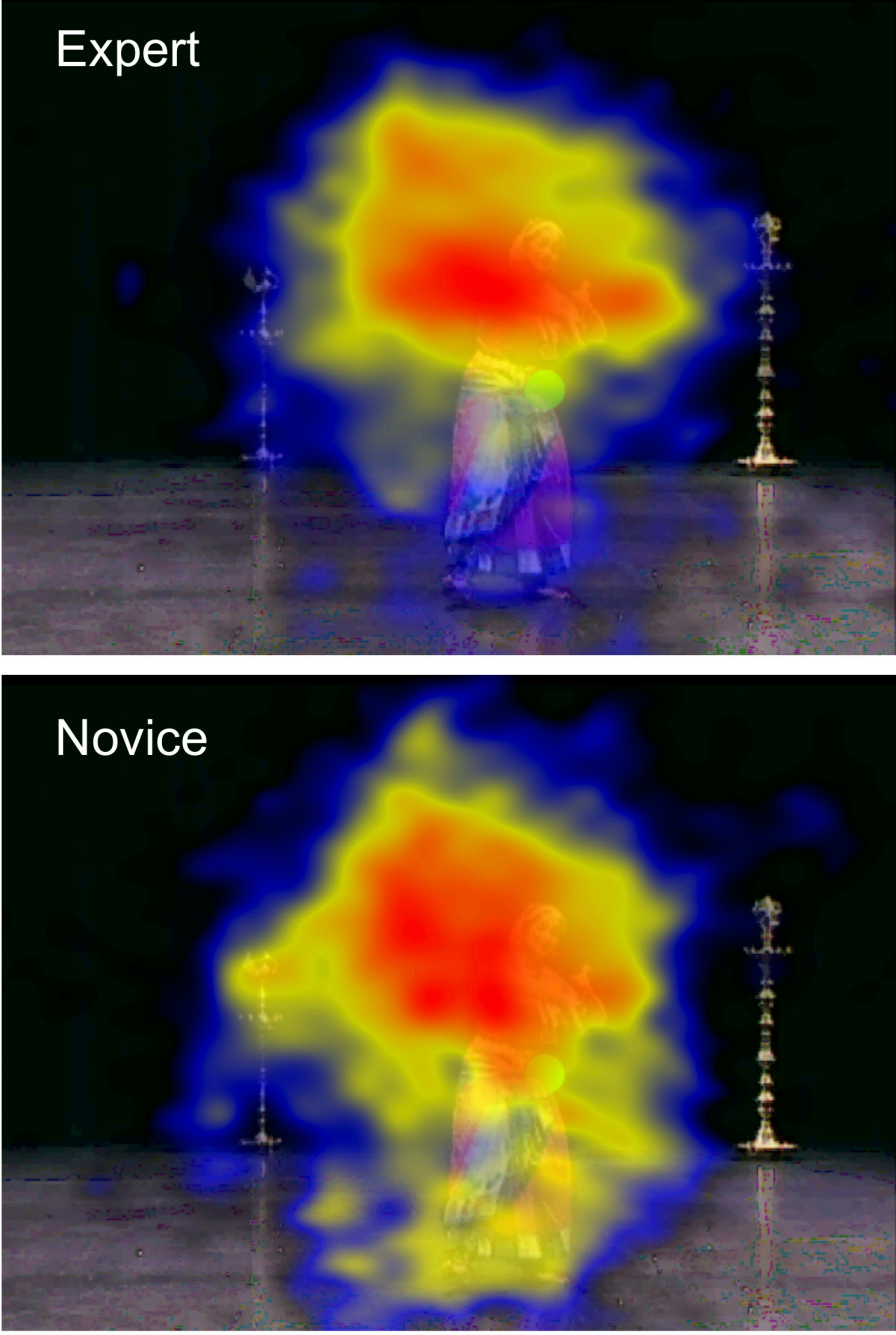
Heat maps showing the relative dispersion of fixations for experts and novices. Red areas depict higher dwell time; blue depicts lower dwell time.

**Figure 7.**
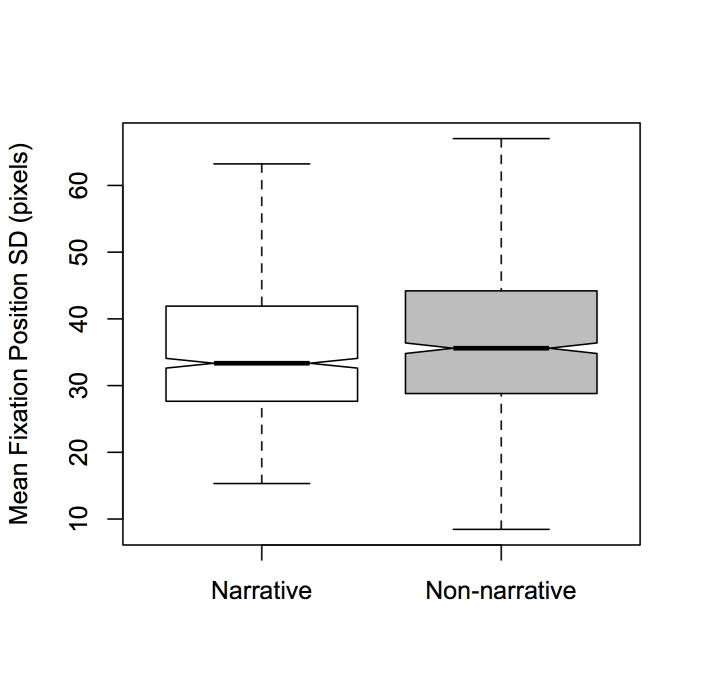
Notched boxplots of mean fixation position SD per time window for *Dance-type* (non-narrative and narrative).

There was a significant main effect of *Expertise* [*F*(1,1890) = 7.074, *p* < 0.01, η² = 0.004]; experts were found to have smaller fixation position SDs compared to novices (see Figure 5). This finding is consistent with the heat maps generated for experts and novices (Figure 6). A significant main effect of *Dance-type* was also found [*F*(1,1890) = 4.693, *p* < 0.05, η² = 0.002]; non-narrative stimuli yielded larger fixation-position SDs than narrative stimuli (see Figure 7). No significant interactions were found between the factors (*F* < 1), and excluding outliers did not affect the significance of the results.

## Discussion

This study explored the extent to which expertise and narrative content influenced the eye movements of people observing Bharatanatyam dance. Two dependent variables were measured, fixation duration and dwell time. A further analysis calculated the dispersion of fixations within 1,000 ms time windows, advancing in increments of 500 ms. Despite the relatively small eta squared effect values, the analyses determined that expert viewers possessed greater attentional similarity than novices: lower fixation-position SDs within a given time period are indicative of greater fixation alignment, and thus increased attentional similarity.

The above was conducted in order to test four related hypotheses. Hypothesis (1)—that novices will have longer fixations than experts—was supported by the data: experts in our study did have shorter fixation durations than novices, suggesting that the inexperienced participants observed the dance videos less efficiently (Figure 2). This result is consistent with previous findings(26) who reported a similar effect in their novice-expert dance study. However, while those findings were concentrated on western contemporary dance(26), our study sought to expand and/or generalize this effect to a very different cultural tradition, i.e. classical dance of southern India.

Hypothesis (2)—that narrative dance versus non-narrative dance will produce differences in eye movements—was not supported by the fixation-duration data, which showed no statistical differences within factor *Dance-type.* This hypothesis was based on previous research that even in the absence of any visual stimuli, contextual information influences oculomotor responses(40). Prior to conducting the experiment, we had speculated that longer fixation durations might occur for the narrative videos as these dances contain richer semantic information, and thus, arguably, require greater attention; however, this was not the case in this instance. That said, a significant effect of *Dance-type* was observed when the dependent variable was fixation position SD (Figure 7). Whether this effect was genuinely cognitive (e.g. involving different attentional resources or processes), or simply due to the dancer moving more in the non-narrative videos is, however, uncertain—dynamic video-contrast analysis(32) would be required in order to answer this point definitively. Be that as it may, given that we are uncertain as to the cause of the effect of *Dance-type* on fixation position SD, the findings above cannot be said to support the conclusions discussed in the Introduction(31); namely, that narrative contexts may contribute less to eye movements than visual features such as motion (i.e. temporal contrast) during free-viewing of videos.

Hypothesis (3)—that greater gaze dwell times will occur in relation to the upper body—was conclusively found to be the case (Figure 4). This result strongly aligns with the findings that, perhaps counter-intuitively, found that the feet of the dancer in their experiment attracted the least fixations(17). Feet are arguably a dancer’s greatest asset(41); it could be considered paradoxically, then, that when observing dance, we seem to spend the least amount of time fixating on this part of the body. That said, an important confound should be mentioned. In scene viewing, gaze direction can be predicted using a combination of saliency and face detection(42), both of which do not depend on whether the scene involves dance. The effects of saliency and face detection significantly bias the upper screen area, in which the face of our dancer was almost invariably located. As a result, no significant conclusions can be made regarding whether dance per se specifically directs attention towards the head.

An interesting finding concerning factor *ROI* was that fixation durations were significantly shorter for the lower versus upper part of the dancer’s body (Figure 3). We confess to being somewhat puzzled by this, although it could be related to the fact that participants spent significantly less time observing the lower part of the dancer’s body (Figure 4). Maybe, observers genuinely find dancers’ legs and feet less interesting (or, at least, informationally impoverished relative to the upper body and head), in which case, when their gaze is drawn downwards, a vertical saccade abruptly directs foveal vision upwards. Recent research supports this conjecture; researchers found that dance communicates group coordination through combined movement dynamics within groups of performers(43). They showed that movement synchrony among a group of performers predicts the aesthetic appreciation of live dance performances(43). They concluded that their findings were in accordance with the evolutionary function of dance in transmitting social signals between people through human movement(43).

Hypothesis (4)—that experts will have greater attentional similarity—was supported by the data: the fixations of the Experts in our study were, in general, more tightly clustered than Novices’ fixations, both spatially and temporally (Figures 5). This is particularly noticeable in the dwell time heat maps in Figure 6: the lower image (novices) clearly has a larger and more diffuse gaze-pattern relative to the upper (experts). This result lends support to the conjecture, referred to in the Introduction, that experienced observers of dance have choreographic schemata stored in long-term memory, which enables them to target their attention towards (shared) salient elements within the dance(26). In turn, this results in a higher degree of attentional similarity between participants, as expressed in fixation-dispersion data. Conversely, inexperienced viewers produce more scattered fixations as they (variously) attempt to make sense of and predict the dancer’s movements. Interestingly, within Bharatanatyam, hand movements are generally considered more important than leg movements, a fact which would have been known to our expert participants, but not novices. Therefore, although the above finding may be due, in part, to dance schemata, the possibility that explicit Bharatanatyam knowledge may have also influenced our results cannot be ruled out.

The fact that our video stimuli were based on examples of Bharatanatyam has both benefits and drawbacks from an experimental perspective. A particular benefit of using excerpts from an authentic Bharatanatyam performance was that they provided the study with a degree of ecological validity—the stimuli were not unduly controlled or contrived, nor stripped of their cultural richness as expressed in the dancer’s costume and the accompanying Carnatic music. It is legitimate to claim, therefore, that our data, however imperfect, were at least produced in response to a real-world phenomenon, and are thus potentially applicable or relatable to other similar global dance practices. For example, future research could similarly examine Kathakali, which, like Bharatanatyam, is a major form of classical Indian dance involving story telling, but which in contrast to Bharatanatyam is predominantly performed by male actor-dancers.

With respect to our study’s limitations, by including only one dancer, factors specific to this individual not controlled for in the experiment may have skewed our results. That said, given the highly codified nature of Bharatanatyam, achieved through multiple years of training, it would seem likely that our findings would be replicated using other dancers. A further potential drawback, previously mentioned, is that our participants were all female. Given that Bharatanatyam is most commonly danced by females, experts with this discipline tend also to be female, if not exclusively so for all practical purposes. Our desire to avoid a gender imbalance between the experts and novices naturally led to the exclusion of male (novice) participants, which may, in itself, impose restrictions on the degree to which our results are generalizable. One further limitation concerns the interdependence of the music and dance—these two crucial factors were not separately manipulated and thus it is possible that there was an undetected interaction between the two, i.e. there may have been factors within the music that interacted with particular gestures, giving rise to specific eye movements. That said, as mentioned previously, all the music was of a similar emotional character and mood, and thus we believe it is unlikely that there were any significant interactions.

Eye-tracking cameras invariably produce a wealth of data that can be analyzed using systems’ proprietary software and/or exported to other analytical packages. In this regard, our decision to concentrate on fixation durations and dispersions, and dwell time may seem unduly restricted: saccade and pupillometric information could, in theory, have also been included in the analysis. Our reason for not doing so was due to the hypotheses we wished to test and their relationship to previous research(26, 17). Certainly, multiple additional statistics could, no doubt, have been included; the extent to which these would have enriched or detracted from the study is, however, open to debate.

## Summary

Our aim was to extend to a broader cultural context a series of findings derived largely from western dance(17, 29), and in this regard the study achieved its main goal. Data consistent with three of our four hypotheses were produced by stimuli consisting of videos of an actual Bharatanatyam dance performance: experts had shorter fixations and greater attentional similarity; greater gaze dwell times occurred predominantly in relation to the upper body. Only one hypothesis achieved limited support: fixation durations showed no difference between narrative and non-narrative videos, whereas fixation dispersion patterns did differ. In sum, the study assists in building a nuanced picture of some of the eye movements associated with Bharatanatyam, and, in so doing, helps pave the way for research investigating other non-western dance forms.

## Ethics and Conflict of Interest

The author(s) declare(s) that the contents of the article are in agreement with the ethics described in http://biblio.unibe.ch/portale/elibrary/BOP/jemr/ethics.html and that there is no conflict of interest regarding the publication of this paper.

## Acknowledgements

This research was supported by funding from the School of the Arts, McMaster University, and the Arts Research Board—Social Sciences and Humanities Research Council (SSHRC), Institutional Grants (SIG) program.

We especially wish to thank Ms. Colleen Tang Poy for her detailed reading of the manuscript, and for her helpful edits and suggestions. Thanks are also due to Mr. James Anthony for his assistance in processing the timewindow information. In addition to the university students who took part in the experiment, special mention should be made of our Bharatanatyam experts without whom this study would have been impossible. We are deeply indebted to them for their input, made possible through years of dedication to dance.
